# Failure to Clear Intermediate Lactate Levels in Ward Patients With Admission Blood Cultures Did Not Increase the Risk of Intensive Care Unit Transfer or In-Hospital Mortality: A Retrospective Cohort Study

**DOI:** 10.7759/cureus.13326

**Published:** 2021-02-13

**Authors:** Roman Dudaryk, Jose R Navas-Blanco, Tanira D Ferreira, Richard H Epstein

**Affiliations:** 1 Anesthesiology, University of Miami, Miami, USA; 2 Pulmonary Medicine, University of Miami, Miami, USA

**Keywords:** sepsis, lactic acid, clinical deterioration, hospital mortality

## Abstract

Introduction

A sepsis bundle instituted by the Centers for Medicare and Medicaid Services, known as SEP-1, mandates remeasuring lactate concentrations in patients with suspected sepsis who have an initial lactate level ≥ 2.0 mmol/L to identify those at risk of mortality or clinical deterioration. However, in the group with an intermediate lactate level (2.0 - 3.9 mmol/L), evidence for the predictive utility for such practice is lacking. The objective of this retrospective cohort study was to evaluate the potential utility of repeating the blood lactate measurement for the premonitory detection of clinical deterioration in patients admitted to a ward with a diagnosis of suspected sepsis and an initial intermediate lactate level.

Methods

Using electronic health records, we retrospectively evaluated all non-hospice adult patients admitted from the emergency department to a ward of an academic medical center between October 1, 2017, and November 30, 2019, in whom a blood culture was obtained on admission as part of their workup for suspected sepsis. Patient demographics, the times and values of lactate concentrations, the occurrence of subsequent intensive care unit (ICU) transfer during the admission, and hospital mortality were determined. We computed the relative risk of ICU transfer (i.e., clinical deterioration) and hospital mortality in patients whose initial lactate was in the intermediate range who failed to reduce their lactate concentration by at least 10% within six hours. We hypothesized that failure to clear the lactate would be associated with an increased risk of ICU transfer and hospital mortality.

Results

We studied 12,157 patients, of whom 25 hospice patients were excluded. Of the remaining 12,132 patients, 1,416 (11.7%) were initially admitted to an intensive care unit, and 10,716 (88.3%) were admitted to a ward. Repeat lactate determinations were performed in 10.7%, 77.1%, and 90.2% of the ward patients with initial normal (< 2.0 mmol/L), intermediate (2.0 - 3.99 mmol/L), and high (≥ 4.0 mmol/L) admission lactate concentrations, respectively. There was no increase in the relative risk of ICU transfer (relative risk [RR] = 0.90, 95% CI, 0.53 - 1.28, P = 0.55) or hospital mortality (RR = 1.23, 95% CI, 0.85 - 1.79, P = 0.27) within the intermediate lactate level group among those whose lactate remained within 10% of the initial value (i.e., no change) or increased by more than 10%, compared to those in whom the level decreased by more than 10%.

Conclusions

Failure to reduce lactate concentrations in ward patients admitted with possible sepsis and an intermediate lactate level was not associated with an increased risk of ICU transfer or mortality. These results call into question the mandate in SEP-1 to routinely repeat the lactate determination in patients presenting with an intermediate concentration.

## Introduction

Sepsis and septic shock are leading causes of morbidity and mortality in the United States. Despite significant progress in recognizing systemic infection, source control of the infection, and timely administration of antimicrobial drugs, the 28-day mortality is 25% [1-3]. Early identification of patients at risk for sepsis is crucial for the timely initiation of treatment and for triage decisions related to intensive care unit (ICU) versus ward admission [3,4]. Recognition and management of sepsis is a focus of policymakers at the state and national level [5]. The Third International Consensus Definitions for Sepsis and Septic Shock Task Force (Sepsis-3) changed the lactate threshold for inadequate organ perfusion from 4.0 to 2.0 mmol/L, substantively expanding the population of patients who meet diagnostic criteria for septic shock. This decision was controversial, as it was based on a Delphi method and the vote was not unanimous.This modification of the threshold for an elevated lactate created a new category of patients with “intermediate lactate levels” (2.0 - 3.9 mmol/L), a group in which management decision-making was not included in previous guidelines [6-8]. The most significant portions of the Sepsis-3 definitions and the Surviving Sepsis Campaign guidelines focus on the management of patients in overt septic shock (i.e., those with elevated lactate and hypotension requiring fluid resuscitation and vasopressors). However, there is limited guidance for patients who have elevated lactate ≥ 2.0 mmol/L but who are not hypotensive on presentation or after an initial fluid bolus in the emergency department (ED) - so-called “cryptic shock” [9,10]. The latest edition of the Surviving Sepsis Campaign recommends repeating lactate levels in patients with intermediate lactate concentrations to identify patients at risk of deterioration [9]. This recommendation to assess lactate clearance became a mandatory component of the sepsis bundle known as SEP-1, a performance measure instituted by the Centers for Medicare and Medicaid Services [11]. Nonetheless, remeasuring lactate concentrations in patients admitted with possible sepsis was found to be the bundle element with the lowest compliance rate [12]. Furthermore, Pepper et al. have argued that evidence supporting the utility of serial lactate measurements to predict deterioration and guide further resuscitation is lacking [13].

In-hospital triage of patients with cryptic shock poses a significant challenge for clinicians because a modestly elevated lactate concentration by itself does not typically warrant admission to an intensive care unit (ICU) [14,15]. The objective of our study was to assess the potential utility of failure to clear lactate from the blood as a marker of subsequent clinical deterioration and mortality in patients admitted to a ward with a diagnosis of possible sepsis and an initial intermediate lactate level. We had two hypotheses related to the outcomes of patients admitted from the ED to a ward with intermediate lactate levels who failed to reduce the lactate level by at least 10% within six hours of the initial measurement. Hypothesis 1 was that failure to clear the lactate would be associated with an increased risk of ICU transfer. Hypothesis 2 was that failure to clear the lactate would be associated with increased in-hospital mortality.

An abstract of this study was presented at the 2020 Florida Association of Anesthesiologists Annual Meeting, June 20, 2020.

## Materials and methods

Data sources and patient cohort

The Institutional Review Board of the University of Miami approved this study with a waiver of consent (IRB#20190845). Inclusion criteria encompassed non-hospice patients ≥ 18 years old admitted through the emergency department ED to the University of Miami Hospital between October 1, 2017, and November 12, 2019, in whom blood cultures were ordered in the ED. Patients admitted to the hospice service were excluded because their treatment is different and palliative care is an exclusion measure from the SEP-1 bundle [11]. Data were obtained from the hospital's electronic health record (Epic Systems Corporation, Verona, WI). We inferred that the ED practitioner entertained a diagnosis of possible sepsis from a blood culture having been obtained in the ED because the entered test order indication was “the patient has a known or suspected infection.” Only considering patients in whom a diagnosis of sepsis was subsequently confirmed would have missed many patients admitted in whom sepsis was in the differential diagnosis and where decision-making related to the initial lactate concentration would apply. As used in previous studies, an order for blood culture was thus used as a surrogate for suspected infection in the workup of patients with suspected sepsis [16]. The STROBE (Strengthening the Reporting of Observational Studies in Epidemiology) checklist for cohort studies was followed in the preparation of this report [17].

Data collected included the patient's age, sex, height, weight, initial admission location (ward or ICU), time of transfer from a ward to an ICU (if occurring), inpatient mortality, time of initial antibiotic administration, time of initial steroid administration, and length of stay. Laboratory values collected while in the ED and during the hospital stay included electrolytes, serum creatinine, partial thromboplastin time, international normalized ratio, white blood cell count, hematocrit, lactate level, albumin, procalcitonin, arterial blood gases, inspired oxygen concentration, and arterial-alveolar gradient. Blood pressure, heart rate, respiratory rate, and vasopressor use (i.e., epinephrine, norepinephrine, vasopressin) were also captured. Patients' admission lactates were characterized according to the value nearest to the time to the time of hospital admission. The lactate concentrations were characterized as: normal (< 2.0 mmol/L), intermediate (2.0 - 3.9 mmol/L), or high (≥ 4.0 mmol/L), or as not measured. Repeat lactate determinations were characterized as decreased if the value was < 90% of the initial measurement, increased if the value was > 10% higher, and unchanged if the absolute value of the difference was within 10% [18].

Outcomes

In patients admitted to a ward with an initial intermediate lactate level, we studied the relative risks of transfer from a ward to an ICU and in-hospital mortality where the most recent repeat lactate level within 6 hours of the initial measurement was unchanged or increased compared to where the repeat lactate level decreased.

Statistical analysis

Relative risks were calculated in RStudio (Version 1.1.456, RStudio, Boston, MA) using the procedure epi.2x2 in the epiR package [19]. Confidence intervals for the incidence of hospital mortality and ICU transfer were determined using the method of batch means, with batching by N = 28 four-week intervals to eliminate potential autocorrelation. Differences in parameters between ward and ICU patients were calculated using the Wilcoxon rank sum test, as nearly all comparisons violated assumptions of normality. The Student's t-test was used for the three parameters that were normally distributed (systolic blood pressure, albumin, and hematocrit). P-values < 0.05 were adjusted for multiple comparisons using the Holm-Bonferroni method, with an adjusted P < 0.05 required to claim statistical significance.

The study was powered to be able to detect a relative risk (RR) of 2.0 for ICU transfer and mortality in patients admitted to a ward with a suspicion of sepsis and an intermediate lactate concentration in whom the repeat lactate was either unchanged or increased (exposure group), compared to patients in whom the lactate decreased (control group). Assuming that 10% of control patients would be transferred to an ICU during their hospitalization, with alpha = 0.05 and power = 0.8, at least 223 patients would be required in each group. Assuming a 7% mortality rate in the control group, 301 patients would be required in each group to similarly detect a relative risk of 2.0 between the exposure and the control group.

## Results

Overall escalation of care and in-hospital mortality

During the study interval, 12,157 patients were admitted to the hospital where a blood culture was obtained in the ED; 25 hospice patients were excluded. Of the remaining 12,132 patients, 10,716 (88.3%) were admitted from the ED to a ward and 1,416 (11.7%) to an ICU. Repeat lactate determinations were performed in 10.7%, 77.1%, and 90.2% of ward patients with normal, intermediate, and high admission lactate concentrations, respectively. Admission antibiotics were administered to 77.3%, 84.6%, and 87.2% of these groups, respectively. The study was sufficiently powered to assess both hypotheses, with 1,316 patients having an initial intermediate lactate concentration in whom the repeat value decreased, and 436 patients in whom the value either did not change or increased.

Clinical characteristics of the patients are presented in Table 1. As expected, patients admitted from the ED to a ward were less ill than those admitted to an ICU from the ED, with significantly better hemodynamic, vital sign, and laboratory values for the vast majority of parameters (Table 1).

**Table 1:** Clinical characteristics of patients admitted with a diagnosis of possible sepsis Abbreviations: ICU, intensive care unit; Q1, first quartile; Q3, third quartile; SpO2, oxygen saturation by pulse oximetry ^a ^Values were from the time closest to arrival in the emergency department. ^b ^P-values < 0.05 were adjusted for the 16 multiple comparisons using the Holm-Bonferroni method.

	Ward Patients	ICU Patients	
Parameter^a^	Median (Q1, Q3)	Median (Q1, Q3)	P-Value^b^
# Patients	10,716	1,416	
Male, %	50.0%	52.1%	0.15
Age, years	63 (51, 75)	68 (57, 79)	<0.001
Body Mass Index, kg∙m^-2^	26.5 (22.8, 31.0)	26.4 (22.8, 31.0)	0.99
SpO2, %	99 (97, 100)	98 (94, 100)	<0.001
Heart Rate, min^-1^	95 (81, 110)	104 (84, 120)	<0.001
Respiratory Rate, min^-1^	18 (16, 20)	20 (17, 25)	<0.001
Temperature, C	36.9 (36.7, 37.3)	36.9 (36.7, 37.4)	0.13
Systolic Blood Pressure, mmHg	130 (114, 147)	120 (97, 145)	<0.001
Hematocrit, %	35.3 (30.2, 39.9)	34.7 (28.9, 40.0)	0.03
White Blood Cells, ×10^-3^	9.7 (6.6, 13.9)	12.0 (7.9, 17.3)	<0.001
Platelets, ×10^-3^	236 (169, 319)	228 (150, 307)	<0.001
Creatinine, mg∙dL^-1^	0.94 (0.72, 1.35)	1.22 (0.83, 1.98)	<0.001
Lactate, mmol∙L^-1^	1.5 (1.1, 2.2)	2.3 (1.5, 3.3)	<0.001
Procalcitonin, ng∙mL^-1^	0.3 (0.1, 1.2)	0.9 (0.2, 5.4)	<0.001
Albumin, g/dL	3.8 (3.3, 4.2)	3.5 (2.9, 4.0)	<0.001
International Normalized Ratio	1.1 (1.0, 1.3)	1.2 (1.1, 1.5)	<0.001
Partial Thromboplastin Time, sec	31.4 (28.1, 35.5)	31.5 (28.1, 36.8)	0.13

**Figure 1:**
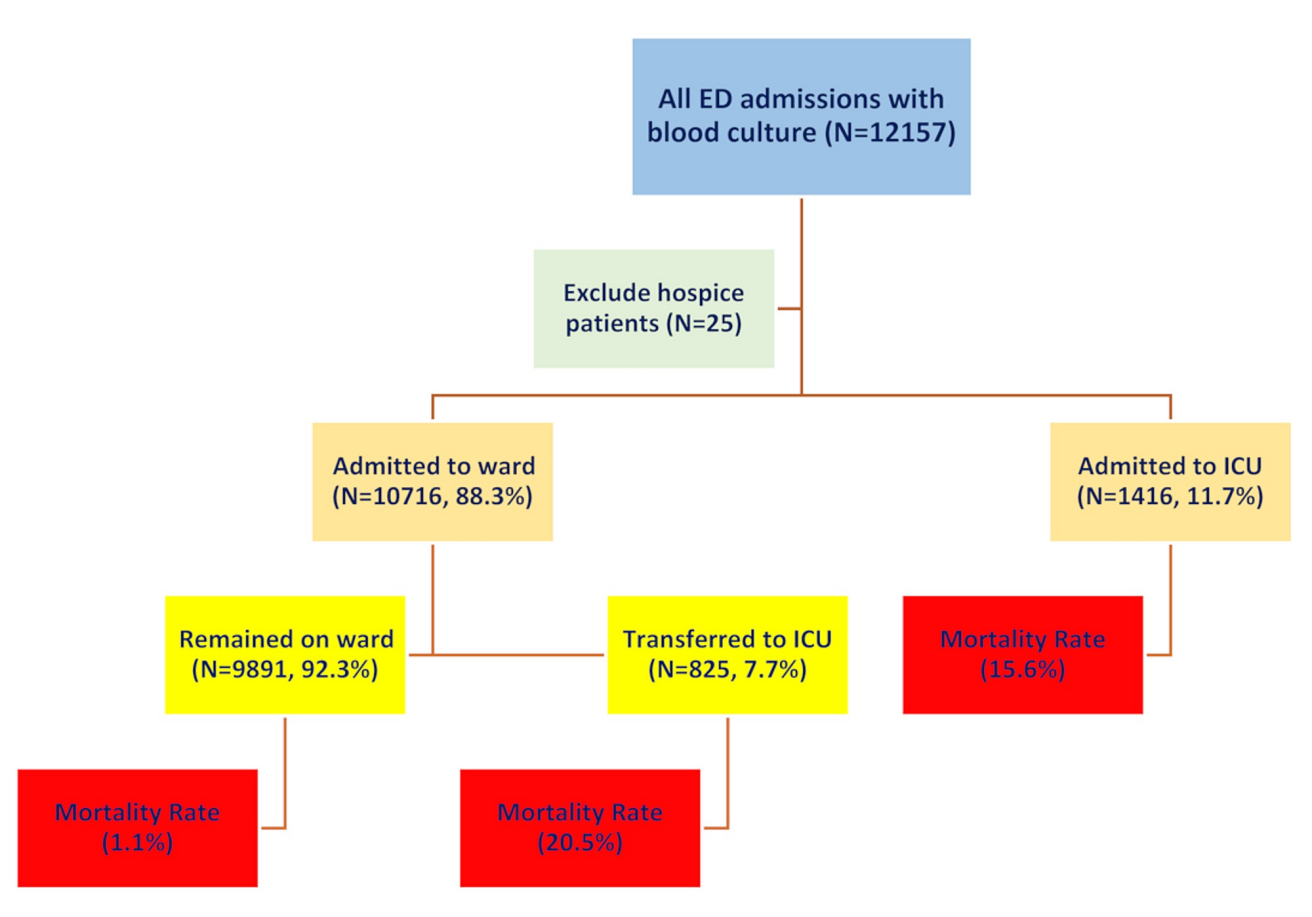
Flow diagram of the population analyzed with in-hospital disposition and mortality rate ED, Emergency Department; ICU, Intensive Care Unit

Summary results related to survival and ICU transfer are presented in Figure 1. The overall hospital mortality rate was 4.2% (95% CI, 3.7% - 4.6%). In-hospital mortality was 2.6% (95% CI 2.3% - 3.0%) in patients initially admitted to a ward and 15.7% (95% CI 13.6% - 17.9%) in patients admitted directly from the ED to an ICU. Among ward patients, 7.7% were subsequently transferred to an ICU. The transferred patients’ mortality rate was 20.5% compared to 15.6% of patients directly admitted to an ICU (RR = 1.31, 95% CI, 1.10 - 1.57; P = 0.003).

Escalation of care and in-hospital mortality in patients admitted to a ward

Among the 10,716 patients admitted initially to a ward, baseline lactate determinations were performed in 86.8%. Transfer rates and mortality were similar between patients whose initial lactate level was in the normal range and those in whom an initial lactate was not measured. Compared to patients with normal admission lactate concentrations, there was a clinically important and statistically significant increased risk of in-hospital mortality in patients with intermediate (RR = 2.65, P < 0.001) and high (RR = 6.69, P < 0.001) lactate concentrations, but not in patients in whom an initial lactate was not measured (Table 2). Compared to patients with normal admission lactate concentrations, there was a clinically important increased risk of in intensive care unit transfer in patients with intermediate (RR = 1.41, P < 0.001) and high (RR = 2.14, P < 0.001) lactate concentrations, but no increased risk in patients in whom an initial lactate was not measured (Table 2).

**Table 2:** Relative risk of in-hospital mortality or intensive care unit transfer based on admission lactate concentration CI, confidence interval; ICU, intensive care unit; RR, relative risk ^a ^Normal: < 2.0 mmol/L; Intermediate: 2.0 – 3.99 mmol/L; High: ≥ 2.0 mmol/L ^b ^P-values < 0.05 were corrected for multiple comparisons using the Holm-Bonferroni method.

		In Hospital Mortality				ICU Transfer		
Admission Lactate^a^	Total	Death	RR vs. Normal Lactate (95% CI)	P-value^b^		Transfer	RR vs. Normal Lactate (95% CI)	P-value^b^
Normal	6327	1.58%				6.75%		
Not Measured	1422	2.11%	1.33 (0.89 – 2.00)	0.16		6.96%	1.03 (0.84 – 2.00)	0.77
Intermediate	2622	4.20%	2.65 (2.03 – 3.47)	<0.001		9.50%	1.41 (1.21 – 3.47)	<0.001
High	388	10.57%	6.69 (4.72 – 9.48)	<0.001		14.43%	2.14 (1.65 – 9.48)	<0.001

Evaluation of hypotheses

Hypothesis 1 was that failure to clear the lactate would be associated with an increased risk of ICU transfer. Among patients admitted to a ward with an intermediate lactate level, there was no increase in the relative risk of transfer to an ICU if the repeat value was unchanged, increased, or did not decrease within six hours, compared to patients in whom the level decreased by > 10% (Table 3). Hypothesis 1 was rejected.

**Table 3:** Direction of change in lactate concentrations measured within 6 hours of the initial value and the relative risk of transfer to the intensive care unit among patients admitted from the emergency department to a ward ICU, intensive care unit; LCL, lower confidence limit; UCL, upper confidence limit; RR, relative risk ^a ^Decreased: > 10% reduction; Unchanged: within 10% of the initial value; Increased: > 10% increase ^b ^Uncorrected P-values were all ≥ 0.05, so no adjustments were made by the Holm-Bonferroni procedure

Lactate Concentration Change^a^	ICU Transfer	No ICU Transfer	Risk	RR vs. Decreased	95% LCL	95% UCL	P-value^b^
Decreased	121	1195	9.2%				
Unchanged	20	211	8.7%	0.94	0.6	1.48	0.79
Increased	16	189	7.8%	0.85	0.51	1.4	0.52
Unchanged or Increased	36	400	8.3%	0.90	0.63	1.28	0.55

Hypothesis 2 was that failure to clear the lactate would be associated with increased in-hospital mortality. Among patients admitted to a ward with an intermediate lactate level, there here was no increase in the relative risk of hospital mortality if the repeat value was unchanged, increased, or did not decrease within six hours, compared to patients in whom the level decreased by at least 10% (Table 4). Hypothesis 2 was rejected.

**Table 4:** Direction of change in lactate concentrations measured within six hours of the initial value and the incidence of inpatient mortality among patients admitted from the emergency department to a ward h, hours; ICU, intensive care unit; LCL, lower confidence limit; UCL, upper confidence limit; RR, relative risk ^a^ Decreased: > 10% reduction; Unchanged: within 10% of the initial value; Increased: > 10% increase ^b^ Uncorrected P-values were all ≥ 0.05, so no adjustments were made by the Holm-Bonferroni procedure

Lactate Concentration Change^a^	Died	Survived	Risk	RR vs. Decreased	95% LCL	95% UCL	P-value^b^
Decreased	88	1228	6.7%				
Unchanged	16	215	6.9%	1.04	0.62	1.73	0.89
Increased	20	185	9.8%	1.46	0.92	2.32	0.11
Unchanged or Increased	36	400	8.3%	1.23	0.85	1.79	0.27

## Discussion

Intermediate or high admission lactate concentrations in patients admitted to a ward with a diagnosis of possible sepsis were associated with an increased relative risk of ICU transfer and hospital mortality compared to patients admitted with possible sepsis who had normal lactate concentrations. However, there was no association between a failure to clear the lactate by more than 10% within six hours and the relative risk of either escalation of care or mortality. Although our results support recommendations to measure the lactate concentration in patients admitted with a diagnosis of possible sepsis at the time of admission for overall risk assessment, routinely repeating the lactate determination in all patients with an initial intermediate lactate was not informative. Thus, our data do not support the mandate within SEP-1 to perform reassessments in all such patients. Although reporting under SEP-1 only applies to patients who ultimately were determined to have sepsis, the practical result of the mandate is that all patients with lactate values ≥ 2.0 mmol/L need to have a repeat value drawn because one will not know in advance which patients will have a final diagnosis of sepsis. Selectively repeating the lactate in patients with an initial intermediate lactate level thus would create a risk of failing the SEP-1 measure because all metric elements are required to pass.

We emphasize that our findings of lack of utility for repeating the lactate are restricted to patients who were sufficiently stable to be admitted to a ward and who have intermediate lactate levels. There are too many patients admitted to a ward with intermediate lactate concentration for them to be admitted to an ICU, but more extensive surveillance should be considered, given their increased risk of deterioration compared to patients with normal admission lactate levels.

We were underpowered to assess the utility of such repeat measurements in ward patients whose lactates were initially in the high range. There are no current recommendations to follow lactates routinely in patients whose initial values are normal, and, indeed, this was seldom performed at the hospital studied. Presumably, patients with normal lactates had a repeat test based on clinical indications. Our findings should not be generalized to patients admitted from the ED directly to an ICU because those patients often are in septic shock (e.g., requiring vasopressor or ventilator support) or are judged to be at high risk of deterioration. Our study should not be interpreted to mean that follow-up lactates should not be performed in any ward patient; rather, this decision should be driven by clinical considerations.

Persistent hyperlactatemia or lack of lactate clearance in patients with sepsis or septic shock has been associated with increased in-hospital morbidity and mortality [4,20-22]. In a 10-year retrospective cohort analysis, Rhee et al. determined that the rate of lactate measurement among hospitalizations for the suspicion of sepsis increased significantly from 11% to 48%, although one-third of patients did not have a lactate level measured and were found to have high hospital mortality rate and length of stay. Further multivariate analysis revealed hospital-onset sepsis and hospitalization in a non-medical service to be the most significant factors associated with this practice (P < 0.001) [23]. 

Hyperlactatemia has been widely used for prognostication of sepsis, given its ability to identify hypoperfusion and metabolic stress states. Previously, persistent hyperlactatemia or lack of lactate clearance in patients with sepsis or septic shock was adopted as a sole biochemical marker of resuscitation [4,20-22]. Many believe that this approach is too simplistic and does not account for other reasons responsible for a decreased lactate clearance other than hypoperfusion. Beta-adrenergic activation and excessive pyruvate production due to a stress response are regarded as common causes of lactate elevation in patients with sepsis. Moreover, some believe that a moderately elevated lactate is evidence of a proper physiologic response, reflecting an appropriate level of endogenous catecholamines and providing energy substrate [24]. As noted by Marik, repeating lactate levels is labor-intensive and failure of the SEP-1 bundle from not repeating the measurement is not associated with a worse outcome [24]. Our finding that failure to clear to lactate in ward patients admitted with an intermediate lactate level is not associated with either clinical deterioration or mortality is consistent with such observations [24].

Central venous oxygen saturation was advocated in a previous edition of the sepsis guidelines as a marker of tissue oxygen delivery and resuscitation target. However, several large randomized trials conclusively demonstrated that it did not improve outcomes of early goal-directed therapy leading to a recommendation against routine use of central venous oxygen saturation monitoring [25,26]. Of interest, Jones et al. have compared central venous oxygen saturation with serial lactate measurement in early sepsis, demonstrating non-inferiority of the later [18].

Limitations

First, this was a non-randomized, single-center, retrospective trial. Despite having an adequate sample size to assess the relationship between failure to clear the lactate and either an increased risk of ICU transfer or in-hospital death among patients admitted to a ward with an intermediate lactate level, the application of the results from this study would require further confirmation in large multicenter trials. Although randomization to performing or not performing a follow-up lactate in patients presenting with intermediate lactate concentrations, such studies would be challenging since retesting is currently considered a standard of care. Second, the studied hospital has a well-established sepsis care protocol with high adherence, based on the surviving sepsis campaign guidelines [27]. Consequently, its sepsis-related mortality is lower than the overall average estimated from the most recent epidemiologic data [3]. Therefore, our findings may not be generalizable to other practice settings. Finally, because we used blood culture as a surrogate marker for suspected infection rather than a constellation of clinical signs, symptoms, and laboratory data, we could not identify subgroups in which trending lactate might have prognostic value.

## Conclusions

Failure to clear the initial lactate concentration by more than 10% within six hours was not associated with an increased risk of ICU transfer or mortality in patients admitted from the ED to a ward in whom the initial level was in the intermediate range (2.0 - 3.9 mmol/L). Thus, routinely retesting such patients lacked utility in predicting the need for escalation of care or death. Our data suggest that the current mandate in SEP-1 to repeat lactate measurements in all patients presenting with intermediate lactate concentrations should be revisited. Randomized controlled trials are needed to establish the value of following serial lactate concentrations.
